# Age-dependent effects on palate volume and morphology during orthodontic RME treatment

**DOI:** 10.1007/s00784-022-04831-0

**Published:** 2023-01-05

**Authors:** Gero Stefan Michael Kinzinger, Jörg Alexander Lisson, Charlotte Buschhoff, Jan Hourfar

**Affiliations:** Department of Orthodontics, Saarland University, Homburg, Saar Germany

**Keywords:** Rapid maxillary expansion (RME), Palate volume, Palate morphology, Palate ratio, Cast analysis

## Abstract

**Objectives:**

Rapid maxillary expansion (RME) shows different age-dependent effects. It has been shown that RME leads to a parallel expansion prior to the age of 10, while later and especially from the age of 12, a V-shaped expansion happens (transverse, anterior > posterior; horizontal, inferior > superior). However, it is not clear to what extent these effects influence palatal volume and morphology and eventually maxillary functional space. The aim of the present study was to examine possible age-related effects of treatment with a dental anchored RME appliance upon volume and width/height ratio of the anterior and posterior palate.

**Materials and methods:**

Sixty children and adolescents with documented treatment histories after RME were divided into three equal groups according to age at treatment begin (PG 1, < 10 years, *n*=20; PG 2, 10 ≤ 12 years, *n*=20; PG 3, > 12 years, *n*=20). Maxillary dental casts before and after therapy were digitised. Changes in palatal volume were determined using 3D analyses.

**Results:**

In all patients, the palatal volume increases significantly after RME. Older patients experienced smaller increases in total and posterior volume in absolute and percentage terms. The anterior palate volume increases are almost equal in all patients. Since palatal width increases more markedly than palatal height, the width/height ratio always increases. Except for the posterior region in PG 3, its increase is significant in all groups, both anteriorly and posteriorly. After successful RME, the palatal morphology appears normal anteriorly in PG 1, PG 2 and PG 3 and rather steep posteriorly in PG 3.

**Conclusions:**

RME treatment with identical force application causes different, age-dependent effects upon palate volume and morphology. Width changes have a greater influence on palate volume than height changes.

**Clinical relevance:**

It is preferable to use an RME prior to the age of 10 if homogeneous changes of the anterior and posterior palate regarding maxillary symmetry and functional space are desired.

## Introduction

Forced skeletal expansion of the maxilla was first described as “rapid maxillary expansion” (RME) or “rapid palatal expansion” (RPE) by Angell in 1860 [1] and is one of the oldest orthodontic treatment modalities. The expansion of the maxilla addresses skeletally caused constriction [2], which is often associated with crossbites and mandibular side shift. The appliance causes the separation of the maxillary palatine processes and the horizontal laminae of the ossa palatina [3], while the pterygoid processes spread laterally in the caudal region [4].

The therapeutic effects of forced palatal expansion have been thoroughly evaluated in many studies. Those focused primarily on the effects on the median palatal sutures [5–11], the circummaxillary sutures [12] and skeletal and dental effects as well as side effects [13–18]. Also, rhinological [19–22], urological [23] and effects on tongue position and airway [24, 25] have been described. In orthodontic therapy, however, the effects on the tooth-bearing palate and the influence of different types of anchorage as well as age dependency are of particular interest.

Kinzinger et al. [26] were the first to analyse the effects of forced skeletal expansion on the morphology of the tooth-bearing palate depending on dentition stages. They demonstrated that the therapeutic effects of RME on palatal morphology vary. The authors concluded that an RME should be performed in the early mixed dentition if a parallel expansion of the palate is desired, since in later dentition stages, maxillary expansion tends to occur V-shaped. In a combined model and CBCT analysis [27], the authors then subdivided the patient population according to chronological age. Width, height and depth measurements of dental casts as well as corresponding CBCT analyses showed that before the age of 10, a parallel transverse expansion occurs, whereas later and especially from the age of 12 onwards, a more V-shaped (transverse, anterior > posterior; horizontal, inferior > superior) and less expressed transverse expansion happens after RME treatment. They concluded that the treatment success of RME depends on the age of the patients at treatment initiation. However, it remained unclear whether these effects also affect palatal morphology and volume and thus the functional space.

The study addressed the following questions:

- Are changes after RME upon palate volume and morphology age-related?
- Can changes of palate volume and morphology be metrically recorded and qualitatively described?
- Is it possible to distinguish changes after RME between anterior and posterior regions of the palate?

## Materials and methods

Of *n*=83 patients who received treatment with a dentally anchored RME appliance between 2015 and 2021, *n*=60 patients (36 female, 24 male) were selected. The selection happened according to the mean frequency and amount of hyrax screw activation. This procedure eliminated activation as a confounding factor.

Other inclusion criteria were:

- No previous orthodontic treatment
- Caucasian origin, based upon visual inspection
- Pronounced transverse upper arch constriction of ≥3 mm according to Pont [28]
- Unilateral or bilateral crossbite
- Existence of high-quality corresponding plaster casts prior to treatment (T_1_) and immediately after removal of the RME appliance (T_2_)

**Fig. 1:**
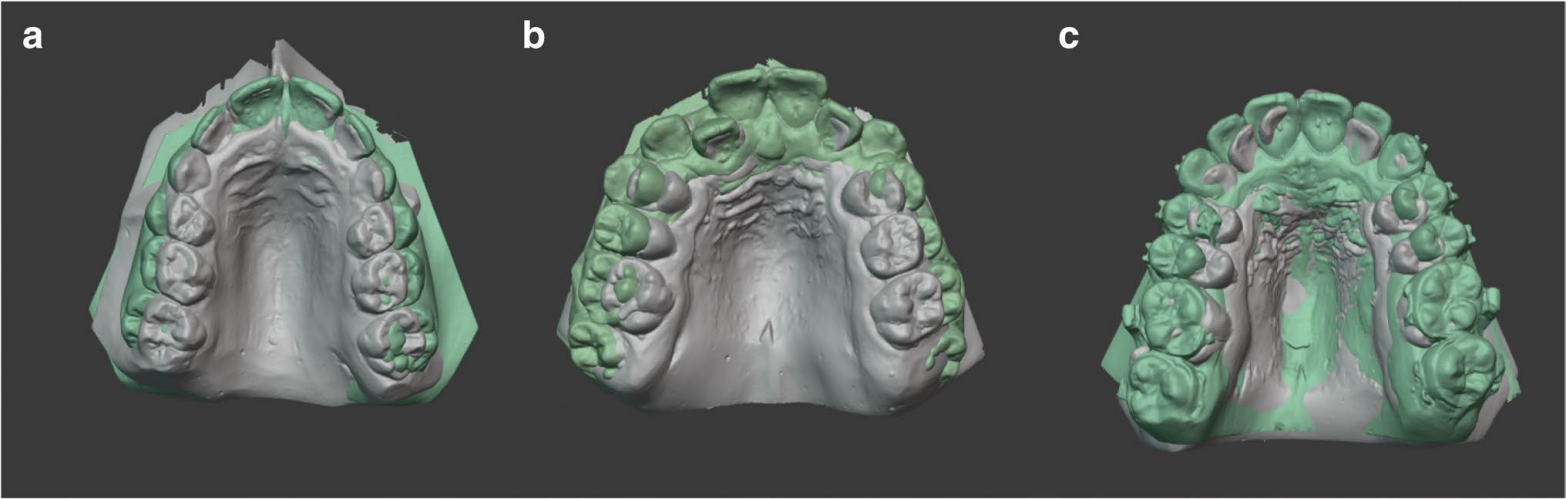
**a–c** (left to right) Treatment examples from PG 1 (**a**), PG 2 (**b**) and PG 3 (**c**): model superimposition at T_1_ (grey) before and T_2_ (green) after palatal expansion

The division into three equal sized (*n*=20) patient groups (Fig. 1a–c) was done according to patient age at treatment begin: PG 1 with patients < 10 years, PG 2 with patients 10 < 12 years and PG 3 with patients ≥ 12 years. The youngest patient was 7.28 and the oldest 16.45 years old at T_0_. The mean age was 11.33 ± 2.60 years (PG 1, 8.57 ± 0.81 years; PG 2, 10.94 ± 0.63 years; PG 3, 14.43 ± 1.41 years).

The RME appliance remained in situ for mean 6.13 ± 1.50 months (PG 1, 6.06 ± 1.69 months; PG 2, 6.26 ± 1.25 months; PG 3, 6.07 ± 1.60 months). The assignment of *n*=60 out of *n*=83 patients depended on the extent of hyrax screw activations. These should be as identical as possible for optimal comparison of therapeutic effects. The average screw activation was 25 times, resulting in a maximum screw expansion of 5 mm. All data on patient age and gender, wearing time, screw activation and the severity of the crossbite and mandibular side shift are shown in Table 1.

**Table 1:** Patients: number (*n*), age, gender, average wearing time of the RME, average number of hyrax screw activations, crossbite and mandibular deviation for the total patient group (all patients) and the patient groups PG 1, PG 2 and PG 3

Patients		All patients	PG 1	PG2	PG 3
Number (*n*)	*n*	60	20	20	20
Age (years)	(M ± SD)	11.33 ± 2.60	8.57 ± 0.81	10.94 ± 0.63	14.43± 1.41
Gender (m/f)	*n*	24 / 36	9 / 11	4 / 16	11 / 9
RME wearing time (months)	(M ± SD)	6.13 ± 1.50	6.06 ± 1.69	6.26 ± 1.25	6.07 ± 1.60
Number of hyrax screw activations	*n*	25.15 ± 5.37	25.10 ± 6.4	25.20 ± 5.0	25.15 ± 4.83
Crossbite On both sides/only right/only left	*n*	60 29/21/10	20 9/8/3	20 10/5/5	20 10/8/2
Mandibular deviation None/right/left	*n*	33/19/8	9/8/3	12/5/3	12/6/2

Mean (M) and standard deviation (SD)

### RME appliance

**Fig. 2:**
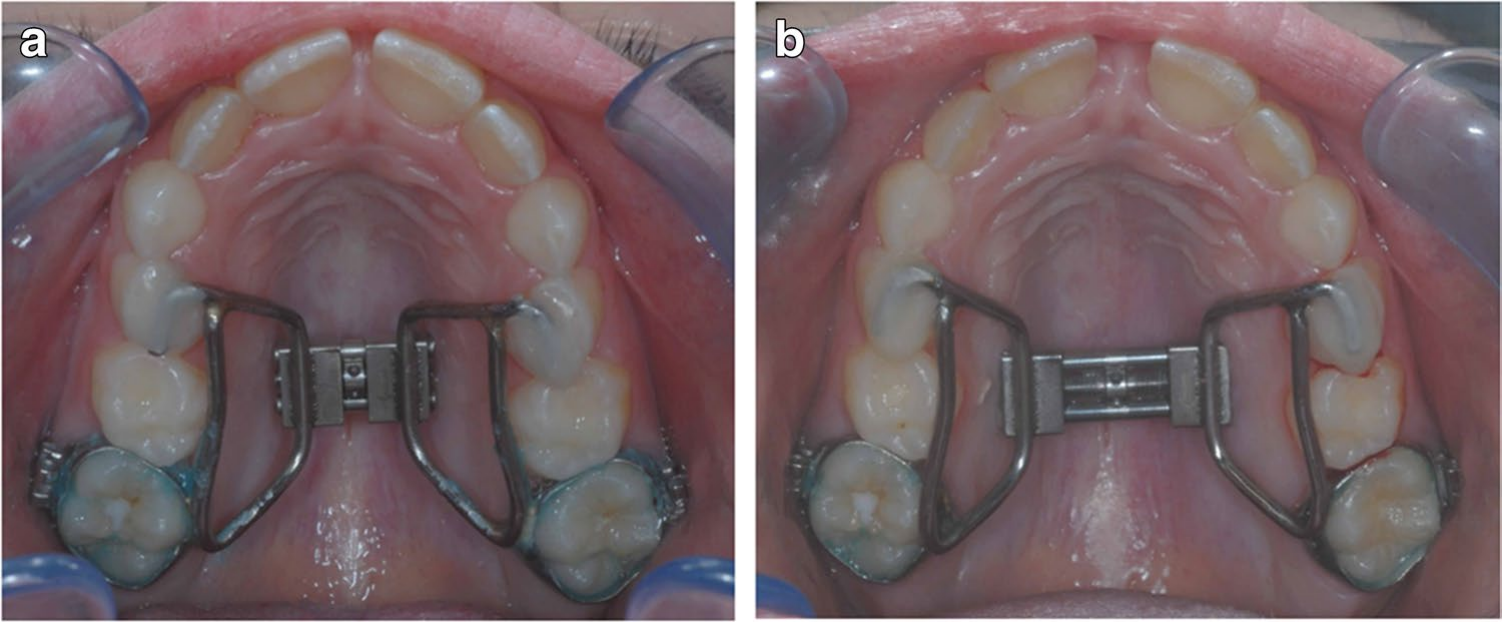
**a** and **b** (left to right) Patient example with RME before (**a**) and after hyrax screw activation (**b**). The appliance is anchored anteriorly with temporary attachment including two occlusal rests on the first deciduous molars and posteriorly with two conventional bands on the first permanent molars

An RME appliance with solely dental anchorage was used in all patients to ensure comparability with other studies. This appliance had a hyrax screw (palatal screw type S, Forestadent, Pforzheim, Germany, lift height 0.2 mm) and was fixed with two bands on the first molars and with two occlusal rests on the first premolars or deciduous molars, respectively (Fig. 2a and b). The activation sequence was twice daily until the targeted transverse expansion including moderate overcorrection was achieved. The appliance then remained passively in situ for approximately 6 months.

### Dental casts

Measurements were performed on 120 dental casts (40 models each from PG 1–3) at:

- T_1_: before forced skeletal expansion of the maxillary complex and at
- T_2_: immediately after removal of the appliance

The impressions were made using rimlock trays and alginate from Kaniedenta (Yellow Print Alginate, Kaniedenta, Herford, Germany). The impressions were cast with plaster (Kanistone Classic, Hartgips type 3, Kaniedenta, Herford, Germany) and trimmed three dimensionally. The orthoX® scan 3D model scanner (Dentaurum, Ispringen, Germany) was used to digitise the plaster model (accuracy of <20 μm with a scan time of 45 s per model). The 3D data sets of the models were optimised with OnxyCeph® 3TM (Image Instruments GmbH, Chemnitz, Germany) and exported as an STL file. The model analysis was then carried out with the software 3D-Tool-Free (3D-Tool-GmbH & Co. KG, Weinheim, Germany).

### Model analysis

The plaster models were used for palate volume measurements according to Wriedt et al. [29]. The digital models were used to measure distances and to calculate quotients as described below.

### Palatal volume

**Fig. 3:**
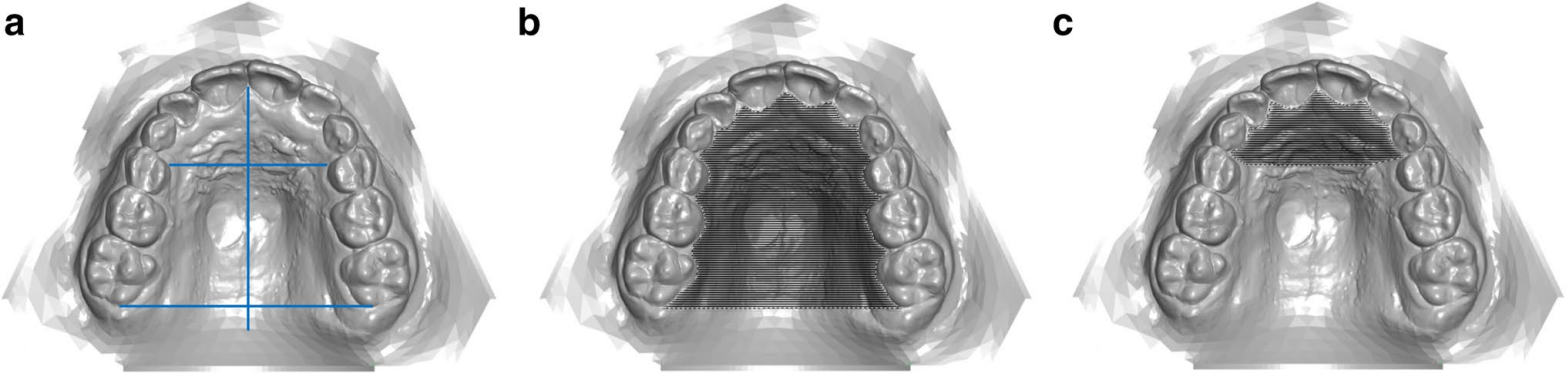
**a**–**c** (left to right) Perpendicular to the raphe median line, a division into an anterior and posterior palatal region is created by a division at the level of the third pair of palatal rugae (**a**). Crosshatched areas: determination of total (**b**) and anterior palatal volume (**c**). The posterior palatal volume was calculated by subtracting the anterior volume from the total volume

The palatal volume was determined by filler quantity measurement, using a modified method according to Wriedt et al. [29]. The palatal volume was divided into an anterior and posterior section to achieve comparable values between T_1_ and T_2_. On each model, the raphe median line, the centre of the third palatal rugae and the distal surfaces of the two first molars were marked first. A perpendicular was drawn from the centre of the third palatal rugae to the raphe median line, and this was extended to the gingival border of the adjacent teeth in the first and second quadrants. Similarly, an interdental dorsal junction line of the first molars was drawn. After determining these anterior and posterior sagittal boundaries on the models, the most coronal point of the gingival margin was marked dental as the horizontal boundary in each case, and the special filler was entered in two measurement series. The filler quantity was first used to calculate the mass of the total palatal volume, and then the anterior palatal volume was determined. The posterior palatal volume was calculated by subtraction [total volume] – [anterior volume]. Both absolute and percentage volume changes were investigated using a ratio: the a/p ratio of the volume is used to determine the expansion pattern (a/p, <1 = inverse V-shaped/delta-shaped; 0 = parallel, >1 = V-shaped/triangular) (Fig. 3a–c).

### Palatal w/h ratio

**Fig. 4:**
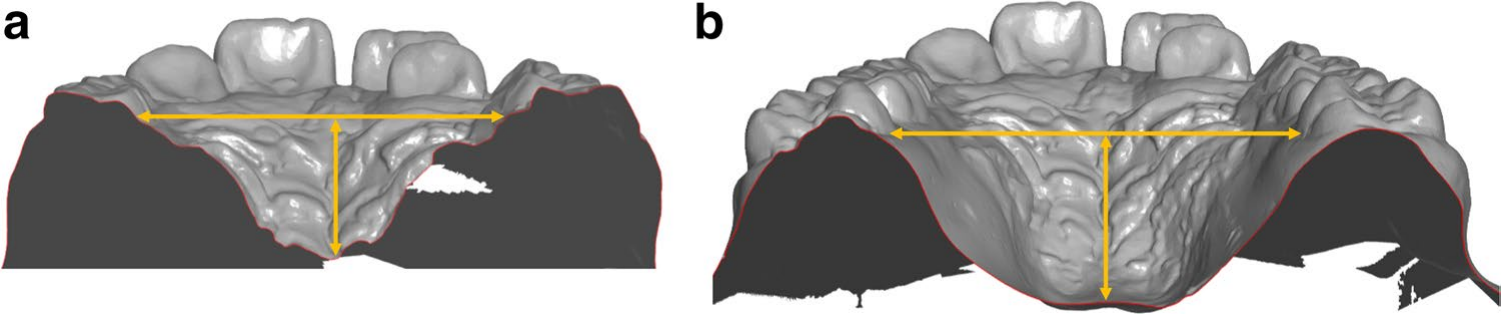
**a** and **b** (left to right) Calculation of anterior and posterior palatal quotients: the gingival width was measured at the level of the landmarks according to Pont [28] anteriorly at the first deciduous molars or premolars (**a**) and posteriorly at the first permanent molars (**b**). The median height was measured perpendicularly between the connecting line and the raphe median line anteriorly (**a**) and posteriorly (**b**)

The method described by Markwardt [30] was modified to assess anterior and posterior palatal shape changes through ratios of distance measurements. The width was measured gingivally, anteriorly and posteriorly at the level of the landmarks according to Pont [28] at first deciduous molars or premolars and at first molars. The anterior and posterior median heights were measured starting from this gingival plane perpendicular to the raphe median line. The ratio of width and height (w/h) allows classification into steep palate (anterior up to 2, posterior up to 2.5), normal palate (anterior 2.1 to 2.9, posterior 2.6 to 3.4) and flat palate (anterior from 3, posterior from 3.5). In addition, the a/p ratio of the gingival plane width was calculated to determine the expansion pattern (values <1 = inverse V-shaped/delta-shaped; 0 = parallel, >1 = V-shaped/triangular) (Fig. 4a and b).

### Statistical evaluation and error of the method

Data were entered in spreadsheet software (Excel®, Microsoft Corporation, Redmond, USA) on a computer with Microsoft® Windows 10 operating system (Microsoft Corporation Redmond, USA) and subsequently imported and analysed in statistical software (SPSS® 23, Armonk, NY, USA) for Windows® (Microsoft Corporation). Normal distribution was tested visually and with the Shapiro–Wilk test. Treatment-associated changes in variables were analysed using the linked *t* test for intra-group comparisons and the independent *t* test for inter-group comparisons. Mean and standard deviation were reported for each variable. Statistical significance was assumed at *p* values < 0.05.

To determine the combined method error (ME) according to Dahlberg [31], 25% of the models randomly selected for this purpose were measured again by the same examiner after a recall-free period of 3 months. The respective method error for volume and angle measurement was calculated with the formula ME =√(∑*d*2/2*n*), where *d* is the difference between two measurement results and *n* is the number of duplicate measurements. The ME was <1 for all measurements in the present study (volume 0.70 cm^3^, width 0.56 mm, height 0.52 mm).

## Results

### Palatal volume

A significant volume increase occurred in all patients after RME. Age-dependent, the initial volume is in PG 1 < PG 2 < PG 3. Contrary to that, the volume increase is both absolute and posterior in PG 1 > PG 2 > PG 3: PG 1 total increase ∆1.75 ± 0.43 cm^3^, posterior ∆1.09 ± 0.42 cm^3^; PG 2 total increase ∆1.68 ± 0.59 cm^3^, posterior ∆1.06 ± 0.58 cm^3^; and PG 3 total increase ∆1.28 ± 0.31 cm^3^, posterior ∆0.58 ± 0.39 cm^3^. Other than that, the anterior volume increases almost similarly in all groups: PG 1 ∆0.65 ± 0.13 cm^3^, PG 2 ∆0.62 ± 0.22 cm^3^ and PG 3 ∆0.70 ± 0.29 cm^3^ (Table 2).

**Table 2:** Palatal volume: total, anterior and posterior

Measurement [mm^3^]	All patients				*p* inter
	T_1_ (M ± SD) 95% CI (LB, UB)	T_2_ (M ± SD) 95% CI (LB, UB)	ΔT_2_–T_1_ (M ± SD) 95% CI (LB, UB)	*p* (intra)	PG 1 vs PG 2 PG1 vs PG 3 PG 2 vs PG 3
Volume total	8.70 ± 1.73 8.25, 9.15	10.27 ± 1.72 9.83, 10.71	1.57 ± 0.50 1.44, 1.70	< 0.001 ^***^	
Volume anterior	1.33 ± 0.50 1.20, 1.46	1.99 ± 0.59 1.84, 2.14	0.66 ± 0.22 0.60, 0.71	< 0.001 ^***^	
Volume posterior	7.37 ± 1.53 6.97, 7.76	8.28 ± 1.51 7.89, 8.67	0.91 ± 0.52 0.78, 1.05	< 0.001 ^***^	
PG 1					
Volume total	8.18 ± 1.85 7.31, 9.04	9.92 ± 2.06 8.96, 10.89	1.75 ± 0.43 1.54, 1.95	< 0.001 ^***^	0.893 ^NS^ 0.006^**^ 0.021^*^
Volume anterior	1.22 ± 0.18 1.13, 1.30	1.87 ± 0.24 1.76, 1.98	0.65 ± 0.13 0.59, 0.72	< 0.001 ^***^	0.891 ^NS^ 0.806 ^NS^ 0.527 ^NS^
Volume posterior	6.96 ± 1.78 6.13, 7.79	8.05 ± 1.98 7.13, 8.98	1.09 ± 0.42 0.90, 1.29	< 0.001 ^***^	0.972 ^NS^ 0.003^**^ 0.006^**^
PG 2					
Volume total	8.50 ± 1.70 7.71, 9.30	10.18 ± 1.70 9.39, 10.98	1.68 ± 0.59 1.40, 1.96	< 0.001 ^***^	0.893 ^NS^ 0.006^**^ 0.021^*^
Volume anterior	1.37 ± 0.58 1.09, 1.64	1.99 ± 0.71 1.65, 2.32	0.62 ± 0.22 0.52, 0.72	< 0.001 ^***^	0.891 ^NS^ 0.806 ^NS^ 0.527 ^NS^
Volume posterior	7.14 ± 1.43 6.47, 7.81	8.20 ± 1.41 7.54, 8.86	1.06 ± 0.58 0.79, 1.33	< 0.001 ^***^	0.972 ^NS^ 0.003^**^ 0.006^**^
PG 3					
Volume total	9.43 ± 1.45 8.75, 10.11	10.71 ± 1.29 10.10, 11.31	1.28 ± 0.31 1.14, 1.42	< 0.001 ^***^	0.893 ^NS^ 0.006^**^ 0.021^*^
Volume anterior	1.42 ± 0.63 1.12, 1.71	2.11 ± 0.71 1.78, 2.44	0.70 ± 0.29 0.56, 0.83	< 0.001 ^***^	0.891 ^NS^ 0.806 ^NS^ 0.527 ^NS^
Volume posterior	8.01 ± 1.18 7.46, 8.56	8.598 ± 1.01 8.12, 9.06	0.58 ± 0.39 0.40, 0.76	< 0.001 ^***^	0.972 ^NS^ 0.003^**^ 0.006^**^

Mean (M), standard deviation (SD), confidence intervals (CI) and significance levels. *NS*, not significant; ^*^p < 0.05; ^**^p<0.01; ^***^p<0.001. Patient groups PG 1, PG 2 and PG 3

Relative to the initial volume, the total volume increased by 22.05 ± 5.84% in PG 1, by 20.75 ± 9.55% in PG 2 and by 14.18 ± 4.95% in PG 3. When distinguished between anterior and posterior, the percentual increase occurred anteriorly rather similar: 54.80 ± 14.37% in PG 1, 49.32 ± 22.54% in PG 2 and 55.61 ± 28.34% in PG 3. There were, however, differences in the increase in the posterior region. While this was 16.24 ± 6.38% in PG 1 and 15.78 ± 10.12% in PG 2, it was only 7.89 ± 6.18% in PG 3.

Significant differences thus exist between PG 1 and PG 3, and between PG 2 and PG 3 in total volume and posterior volume, but not in anterior volume increase. The a/p ratio of the absolute volume further indicates a V-shaped opening in PG 3 (1.21) and a parallel to trapezoidal opening in PG 1 (0.60) and PG 2 (0.58) (Table 3).

**Table 3:** Palatal volume ratio: total, anterior and posterior

Ratio	All patients	PG 1	PG2	PG 3	*p* inter
	ΔT_2_–T_1_ (M ± SD) 95% CI (LB, UB)	ΔT_2_–T_1_ (M ± SD) 95% CI (LB, UB)	ΔT_2_–T_1_ (M ± SD) 95% CI (LB, UB)	ΔT_2_–T_1_ (M ± SD) 95% CI (LB, UB)	PG 1 vs PG 2 PG1 vs PG 3 PG 2 vs PG 3
Volume total					
Ratio total T_2_–T_1_	1.19 ± 0.08 1.17, 1.21	1.22 ± 0.06 1.19, 1.25	1.21 ± 0.10 1.16, 1.25	1.14 ± 0.05 1.12, 1.16	0.830 ^NS^ 0.002^**^ 0.013^*^
Ratio total T_2_–T_1_ in %	18.99 ± 7.77 16.99, 21.00	22.05 ± 5.84 19.32, 24.79	20.75 ± 9.55 16.28, 25.22	14.18 ± 4.95 11.86, 16.49	0.830 ^NS^ 0.002^**^ 0.013^*^
Volume anterior					
Ratio anterior T_2_–T_1_	1.53 ± 0.22 1.47, 1.59	1.53 ± 0.22 1.47, 1.59	1.49 ± 0.23 1.39, 1.60	1.56 ± 0.28 1.42, 1.69	0.723 ^NS^ 0.993 ^NS^ 0.652 ^NS^
Ratio anterior T_2_–T_1_ in %	53.24 ± 22.29 47.49, 59.00	53.24 ± 22.29 47.49, 59.00	49.32 ± 22.54 38.77, 59.87	55.61 ± 28.34 42.35, 68.88	0.723 ^NS^ 0.993 ^NS^ 0.652 ^NS^
Volume posterior					
Ratio posterior T_2_–T_1_	1.13 ± 0.09 1.11, 1.16	1.16 ± 0.06 1.13, 1.19	1.16 ± 0.10 1.11, 1.21	1.08 ± 0.06 1.05, 1.11	0.981 ^NS^ 0.004^**^ 0.006^**^
Ratio posterior T_2_–T_1_ in %	13.30 ± 8.57 11.09, 15.52	16.24 ± 6.38 13.26, 19.23	15.78 ± 10.12 11.05, 20.52	7.89 ± 6.18 4.99, 10.78	0.981 ^NS^ 0.004^**^ 0.006^**^

The width/height ratio for the anterior and posterior region was calculated (total vault and in percent). Mean (M), standard deviation (SD), confidence intervals (CI) and significance levels. *NS*, not significant; ^*^p < 0.05; ^**^p<0.01; ^***^p<0.001. Patient groups PG 1, PG 2 and PG 3

### Palatal width, height and w/h ratio

A palatal width and height increase occurred in all patients after RME. These increases are always significant with the exception of the height values posteriorly in PG 2 and PG 3.

At the gingival level, the increase in width was greater in PG 1 posteriorly (4.14 ± 1.83 mm) than anteriorly (3.18 ± 1.71 mm). In contrast, this was greater in PG 2 and PG 3 anteriorly than posteriorly (PG 2 anterior 3.93 ± 1.14 mm, posterior 3.73 ± 1.48 mm; PG 3 anterior 3.99 ± 1.77 mm, posterior 2.72 ± 1.66 mm) (Table 4).

**Table 4:** Gingival alveolar width (transverse plane), palatal height (frontal plane)

Measurement [mm]	All patients				*p* inter
	T_1_ (M ± SD) 95% CI (LB, UB)	T_2_ (M ± SD) 95% CI (LB, UB)	ΔT_2_–T_1_ (M ± SD) 95% CI (LB, UB)	*p* (intra)	PG1 vs PG2 PG1 vs PG3 PG2 vs PG3
Width anterior, gingival	24.29 ± 1.40 23.92, 24.65	27.98 ± 1.50 27.60, 28.37	3.70 ± 1.58 3.29, 4.11	< 0.001 ^***^	
Height anterior, median	10.24 ± 1.85 9.77, 10.72	11.02 ± 2.00 10.50, 11.53	0.77 ± 1.19 0.47, 1.08	< 0.001 ^***^	
Width posterior, gingival	31.50 ± 2.35 30.89, 32.11	35.03 ± 2.90 34.28, 35.78	3.53 ± 1.74 3.08, 3.98	< 0.001 ^***^	
Height posterior, median	13.38 ± 2.46 12.74, 14.01	13.77 ± 2.69 13.08, 14.46	0.39 ± 0.99 0.14, 0.65	0.003 ^**^	
PG 1					
Width anterior, gingival	24.31 ± 1.69 23.52, 25.11	27.49 ± 1.56 26.76, 28.22	3.18 ± 1.71 2.38, 3.98	< 0.001 ^***^	0.293 ^NS^ 0.238 ^NS^ 0.991 ^NS^
Height anterior, median	9.90 ± 1.60 9.15, 10.65	10.49 ± 1.75 9.67, 11.31	0.59 ± 1.07 0.09, 1.09	0.023 ^*^	0.781 ^NS^ 0.735 ^NS^ 0.997 ^NS^
Width posterior, gingival	32.01 ± 2.57 30.81, 33.22	36.15 ± 2.92 34.78, 37.52	4.14 ± 1.83 3.28, 4.99	< 0.001 ^***^	0.725 ^NS^ 0.024 ^*^ 0.138 ^NS^
Height posterior, median	11.72 ± 1.52 11.00, 12.43	12.25 ± 1.82 11.40, 13.10	0.53 ± 1.12 0.01, 1.05	0.047 ^*^	0.743 ^NS^ 0.840 ^NS^ 0.984 ^NS^
PG 2					
Width anterior, gingival	24.34 ± 1.15 23.80, 24.88	28.26 ± 1.10 27.75, 28.78	3.93 ± 1.14 3.39, 4.46	< 0.001 ^***^	0.293 ^NS^ 0.238 ^NS^ 0.991 ^NS^
Height anterior, median	10.30 ± 2.23 9.26, 11.34	11.15 ± 2.68 9.90, 12.40	0.85 ± 1.20 0.29, 1.41	0.005 ^**^	0.781 ^NS^ 0.735 ^NS^ 0.997 ^NS^
Width posterior, gingival	31.62 ± 1.51 30.92, 32.33	35.36 ± 1.72 34.55, 36.16	3.73 ± 1.48 3.04, 4.43	< 0.001 ^***^	0.725 ^NS^ 0.024 ^*^ 0.138 ^NS^
Height posterior, median	13.67 ± 3.03 12.25, 15.08	13.96 ± 3.35 12.39, 15.53	0.30 ± 1.01 -0.18, 0.77	0.206 ^NS^	0.743 ^NS^ 0.840 ^NS^ 0.984 ^NS^
PG 3					
Width anterior, gingival	24.21 ± 1.39 23.56, 24.86	28.20 ± 1.72 27.39, 29.00	3.99 ± 1.77 3.16, 4.82	< 0.001 ^***^	0.293 ^NS^ 0.238 ^NS^ 0.991 ^NS^
Height anterior, median	10.53 ± 1.69 9.55, 11.27	11.41 ± 1.30 10.62, 12.47	0.88 ± 1.33 0.25, 1.38	0.008 ^**^	0.781 ^NS^ 0.735 ^NS^ 0.997 ^NS^
Width posterior, gingival	30.85 ± 2.76 29.56, 32.14	33.57 ± 3.31 32.02, 35.12	2.72 ± 1.66 1.94, 3.49	< 0.001 ^***^	0.725 ^NS^ 0.024 ^*^ 0.138 ^NS^
Height posterior, median	14.75 ± 1.53 14.13, 15.35	15.10 ± 1.87 13.35, 15.76	0.35 ± 0.88 -0.08, 0.80	0.089 ^NS^	0.743 ^NS^ 0.840 ^NS^ 0.984 ^NS^

Maxillary widths (in mm) in the anterior (54–64/14–24) and posterior (16–26) regions at the gingival level and palatal heights (in mm) (see also Fig. 4a and b)

Mean (M), standard deviation (SD), confidence intervals (CI) and significance levels. *NS*, not significant; ^*^p < 0.05; ^**^p<0.01; ^***^p<0.001. Patient groups PG 1, PG 2 and PG 3

The a/p ratio of the palatal width changes further indicates a V-shaped opening in PG 3 (1.46), a parallel opening in PG 2 (1.05) and an inverse V-shaped, i.e. trapezoidal opening in PG 1 (0.77).

In PG 1, the median height showed significant increases anteriorly and posteriorly. In PG 2 and PG 3, however, the posterior height change is not significant. In the sagittal, antero-posterior comparison, the median height increases in PG 1 occurred almost equal but in PG 2 and PG 3 much more pronounced anteriorly than posteriorly. There are no significant differences between the groups.

**Fig. 5:**
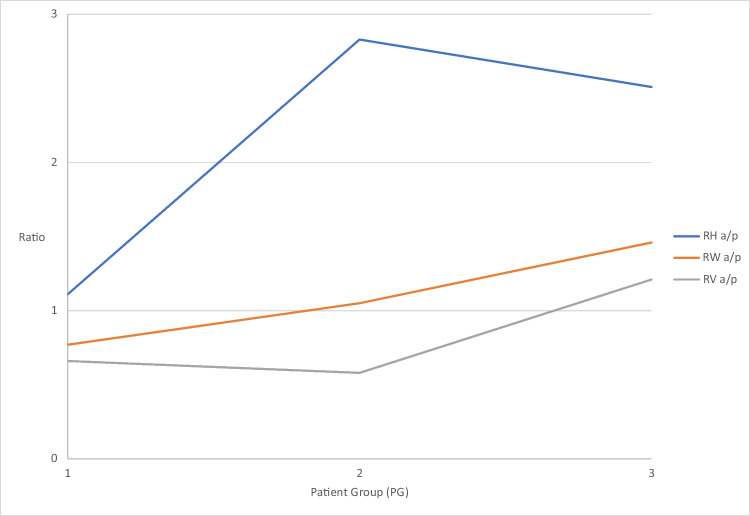
Graphical representation of the a/p ratio T_2_–T_1_ for palatal volume (RV, grey), palatal width (RW, orange) and palatal height (RH, blue) changes

The a/p ratio of the palatal height shows a uniform change only in PG 1 (1.11), whereas the values for PG 2 (2.83) and PG 3 (2.51) show different changes (Fig. 5).

Since the widths increase much more than the heights, the quotient of width and height (w/h) increases everywhere. The changes in the palatal quotient are significant anteriorly and posteriorly within all groups except for the posterior region in PG 3.

When regarding palatal morphology, only the posterior region of PG 3 shows a steep palate after therapy. Otherwise, all other areas of the 3 patient groups show a normal palate (Table 5).add footnote here

**Table 5:** Calculation of anterior and posterior width/height ratio for all groups

Ratio	All patients				*p* inter
	T_1_ (M ± SD) 95% CI (LB, UB)	T_2_ (M ± SD) 95% CI (LB, UB)	ΔT_2_–T_1_ (M ± SD) 95% CI (LB, UB)	*p* (intra)	PG 1 vs PG 2 PG1 vs PG 3 PG 2 vs PG3
Anterior width/height ratio	2.44 ± 0.46 2.33, 2.56	2.61 ± 0.47 2.49, 2.74	0.17 ± 0.31 0.09, 0.25	< 0.001 ^***^	
Posterior width/height ratio	2.44 ± 0.50 2.31, 2.57	2.64 ± 0.55 2.50, 2.78	0.20 ± 0.20 0.15, 0.26	< 0.001 ^***^	
PG 1					
Anterior width/height ratio	2.54 ± 0.50 2.29, 2.76	2.70 ± 0.55 2.44, 2.96	0.18 ± 0.28 0.05, 0.31	< 0.001 ^***^	0.997 ^NS^ 0.952 ^NS^ 0.926 ^NS^
Posterior width/height ratio	2.78 ± 0.47 2.56, 3.00	3.00 ± 0.44 2.80, 3.21	0.22 ± 0.22 0.12, 0.33	< 0.001 ^***^	0.951 ^NS^ 0.460 ^NS^ 0.299 ^NS^
PG2					
Anterior width/height ratio	2.45 ± 0.49 2.23, 2.68	2.64 ± 0.50 2.41, 2.87	0.18 ± 0.30 0.05, 0.32	0.011 ^*^	0.997 ^NS^ 0.952 ^NS^ 0.926 ^NS^
Posterior width/height ratio	2.41 ± 0.50 2.18, 2.65	2.65 ± 0.57 2.39, 2.92	0.24 ± 0.22 0.14, 0.34	< 0.001 ^***^	0.951 ^NS^ 0.460 ^NS^ 0.299 ^NS^
PG3					
Anterior width/height ratio	2.35 ± 0.40 2.17, 2.54	2.50 ± 0.33 2.35, 2.66	0.15 ± 0.35 0.02, 0.31	< 0.001 ^***^	0.997 ^NS^ 0.952 ^NS^ 0.926 ^NS^
Posterior width/height ratio	2.11 ± 0.29 1.98, 2.25	2.26 ± 0.37 2.08, 2.43	0.14 ± 0.16 0.07, 0.22	0.074 ^NS^	0.951 ^NS^ 0.460 ^NS^ 0.299 ^NS^

For measurements, see Table 4. Mean (M), standard deviation (SD), confidence intervals (CI) and significance levels. *NS*, not significant; ^*^p < 0.05; ^**^p<0.01; ^***^p<0.001. Patient groups PG 1, PG 2 and PG 3

## Discussion

### Patients and methods

Male and female patients were pooled for this investigation to increase numbers. There might be gender-specific outcome differences, specifically earlier suture maturation in females than in males [32]. However, those were described for significantly older patients — aged 16 and above — than in the current study. A histologic study by Persson and Thilander [33] did not discover gender-specific differences in suture maturation. The results of this retrospective study show summation effects from natural growth and therapeutic effects. To determine net effects of treatment, natural growth would have to be subtracted from each measurement method. However, measurement data of untreated patients with the same initial findings — pronounced maxillary arch constriction — in a corresponding period of time are neither available from own investigations nor from other growth studies. However, it can be assumed that growth effects during a mean treatment period of 6 months are negligible compared to therapeutic effects provoked by an RME. The appliances also remained passive for retention over a comparable time span in all patients. Furthermore, patients were selected out of a larger collective to ensure that the number of hyrax screw activations was almost identical.

A limitation is that measurements were carried out indirectly on plaster and digital models. Bony structures can only be recorded approximately in this way, as the mucosal thicknesses can vary at different points in time.

### Palatal volume

Established methods described in the literature were modified to assess treatment-related changes of volume and morphology. First, the palate was divided into an anterior and posterior region by horizontal division in the region of the middle of the third pair of palatal rugae. This anatomical structure was selected according to a study by Christou and Kiliaridis [34]. The authors described that although all palatal rugae pairs are subject to growth-related changes, those are least pronounced in the third palatal rugae, making them suitable as a reference area for studies observing a short time span.

Especially with conventional dental anchorage, the alveolar processes bend up during RME [17, 35]. In contrast to Wriedt et al. [29], the palatal volume was only determined up to the gingival margin and not up to the occlusal plane, which minimised the influence of buccal tipping upon measurement. Since the initial palatal volume is smaller in younger than in older patients [36], percentage changes are more meaningful than absolute changes.

### Palatal width, height and w/h ratio

The advantage of using a ratio is that palatal width and height changes can be assessed and compared independent from their initial magnitude. Both width and height increase after RME treatment, with much more pronounced changes in width than in height. The method described by Markwardt [30] for determining a palatal quotient has been modified because it relates its measurements to the area of the second deciduous molars or second premolars. Since the lowest point of the palate is anatomically located in this region and the width is considerably smaller than in the molar region, the values defined by the author for the subdivision into flat, normal and pointed palates could not be adopted for this study. That is why own values were defined for the molar region, based on results of the study by Lione et al. [37] on untreated patients.

Furthermore, a division into an anterior and posterior palate area allows a more differentiated consideration of RME treatment effects, since a preliminary study [27] also indicates the existence of age-dependent a/p differences in changes of the palatal morphology.

Palatal width changes after RME are always significant, the height changes in the anterior region in all groups as well, but in the posterior region only in the youngest patients (PG 1). Both the a/p ratio of the absolute volume and the palatal widths as well as the palatal heights show a coordinated tendency: the changes in palatal width vary from inverse V-shaped, i.e. trapezoidal in PG 1 (0.77) to parallel in PG 2 (1.05) to V-shaped in PG 3 (1.46). The changes in height initially appear uniform in the area of the tooth-bearing palate at PG 1 (1.11). Already in PG 2 (2.83), and also in PG 3 (2.51), a relative imbalance develops with changes in the sagittal plane. This is due to smaller height increases in the posterior region in the older patients from PG 2 and PG 3. The ratio of volume is similar between PG 1 (0.60) and PG 2 (0.58) but changes significantly in PG 3 (1.21). This indicates that width changes have a much greater influence on palate volume increase than height changes.

### Medical significance of the change in palatal volume

The medical significance of the changes in the maxillary palate caused by RME is that decisive influences are exerted on the dentition, the surrounding craniofacial structures and the entire body statics [21, 38–40]. Furthermore, changes in palatal volume and morphology have particular effects on tongue position and airway volume. Iwasaki et al. [25] believe that an increase in total airway volume results from a palatal volume expansion. Ozbek et al. [41] demonstrated significant reductions in the distances between the tongue and palate and between the hyoid bone and mandibular plane after RME in a clinical study, and the new tongue posture was found to be stable during follow-up. This also indicates that RME might be more beneficial in early than in late treatment, especially after 12 years of age. Functional stabilisation of a successful expansion by achieving a physiological swallowing pattern and a physiological tongue position is more likely to occur with a parallel opening of the suture and symmetrical expansion of the tooth-bearing palate. The more V-shaped widening of the suture and palate observed in late treatment is associated with a caudal tongue position and thus leads to an increased risk of relapse due to a lack of functional stabilisation [42].

### Influence of age-related sutural changes

The forces and moments occurring through RME affect not only the maxilla but also the ossa palatina and the processus pterygoidei of the os sphenoidale [12, 13, 43–46]. The tensions are initially concentrated on the anterior palate and then proceed dorsally along the median palatal suture and via the palatine bone to the sphenoid bone [47]. After the opening of the median palatal suture, however, the generated forces do not drop significantly. Thus, the main resistance to palatal expansion cannot lie in the median palatal suture itself but in other maxillary connections [48, 49]. Accordingly, the increasing obliteration tendency or bone density of the median palatal suture is not the only decisive factor for the therapeutic effects of forced skeletal expansion of the maxillary complex [27, 50]. The results of the present and a preliminary study [27] indicate that cascading obliterations of the transverse pterygopalatine and palatomaxillary sutures appear to be decisive for the quality of the expansion (parallel or V-shaped) of the median palatine suture and thus also for the morphological changes of the palate. Kinzinger et al. [27] were able to demonstrate the following age-dependent effects based on CBCT analyses. If, in addition to the median palatal suture, the transverse palatal suture connecting the processus palatini of the maxilla with the laminae horizontales of the ossa palatina and also the paired pterygopalatomaxillary sutures are not or not yet completely obliterated, there is a broad and almost parallel widening of the median palatal suture and a corresponding morphological change in the maxillary palate, particularly in the ventral portion in the area of the palatine processes of the maxilla. If the pterygopalatomaxillary sutures are obliterated while the transverse palatal suture is still open, the opening width of the median palatal suture is comparatively smaller, although the opening mode remains almost parallel. If, however, the pterygopalatomaxillary and palatomaxillary sutures are also obliterated due to patient age, the involved bones behave as one unit under the therapeutic force systems [4]. The result is a centre of resistance shift and dorso-cranial rotation [51], a V-shaped opening of the median palatal suture and a consecutive corresponding influence on the palatal morphology. The effects of the RME appliance on the quality and quantity of the expansion of the median palatal suture are thus influenced by interactions with the transverse palatal sutures. In younger patients, the laminae horizontales of the ossa palatina are involved in addition to the palatine processes of the maxilla, whereas with increasing age and cascading obliterations of the transversely running pterygopalatine and palatomaxillary sutures, only the maxillary bone portions are affected. A change in the position of the centres of rotation and resistance is therefore also the cause of the therapeutically induced, age-dependent changes in palatal morphology and palatal ratio observed in this study.

## Conclusions

The present study was the first to determine the age-dependent effects of forced skeletal expansion of the maxilla upon volume and morphology of the tooth-bearing palate distinguishing between anterior and posterior areas. The results allow the following conclusions:

1. RME treatment with identical force application causes different, age-dependent effects upon palate volume and morphology.
2. Width changes have a greater influence on palate volume than height changes.
3. It is advantageous to use an RME prior to the age of 10 if homogeneous changes of the anterior and posterior tooth-bearing palate regarding volume and morphology are desired.
